# Increased vertebral morphometric fracture in patients with postsurgical hypoparathyroidism despite normal bone mineral density

**DOI:** 10.1186/1472-6823-13-1

**Published:** 2013-01-03

**Authors:** Maira L Mendonça, Francisco A Pereira, Marcello H Nogueira-Barbosa, Lucas M Monsignore, Sara R Teixeira, Plauto CA Watanabe, Lea MZ Maciel, Francisco JA de Paula

**Affiliations:** 1 Department of Internal Medicine, School of Medicine of Ribeirão Preto, University of São Paulo, São Paulo, Brazil; 2 Department of Radiology, School of Dentistry of Ribeirão Preto, University of São Paulo, São Paulo, Brazil; 3 Department of Internal Medicine, School of Medicine of Ribeirão Preto, University of São Paulo, Av. Bandeirantes 3900, Ribeirão Preto, SP, 14049-900, Brazil

**Keywords:** Postsurgical hypoparathyroidism, Osteoporosis, Morphometric fracture, Parathyroid hormone, Panoramic radiography

## Abstract

**Background:**

The mechanism behind parathyroid hormone (PTH) activation of bone remodeling is intimately dependent on the time of exposure of bone cells to hormone levels. Sustained high PTH levels trigger catabolism, while transitory elevations induce anabolism. The effects of hypoparathyroidism (PhPT) on bone are unknown. The objective was to study the impact of PhPT on bone mineral density (BMD), on the frequency of subclinical vertebral fracture and on mandible morphometry.

**Methods:**

The study comprised thirty-three postmenopausal women, 17 controls (CG) and 16 with PhPT (PhPTG) matched for age, weight and height. Bone mineral density (BMD) of lumbar spine, total hip and 1/3 radius, radiographic evaluation of vertebral morphometry, panoramic radiography of the mandible, and biochemical evaluation of mineral metabolism and bone remodeling were evaluated in both groups.

**Results:**

There were no significant differences in lumbar spine or total hip BMD between groups. There was marked heterogeneity of lumbar spine BMD in PhPTG (high = 4, normal = 9, osteopenia = 1, and osteoporosis = 2 patients). BMD was decreased in the 1/3 radius in PhPTG *P < 0.005)*. The PhPTG group exhibited an increased frequency of morphometric vertebral fractures and decreased mandible cortical thickness.

**Conclusion:**

The study suggests that vertebral fragility occurs in PhPT despite normal or even high BMD. The current results encourage further studies to evaluate the use of panoramic radiography in the identification of osteometabolic disorders, such as PhPT and the development of a more physiological treatment for PhPT.

## Background

During this decade a remarkable advance has occurred in the knowledge about the molecular and cellular action of PTH on bone [1,2], whereas little progress has been made regarding the physiological role of PTH secretion in bone mass development and homeostasis [3]. There are few lines of evidence suggesting that individuals with normal bone mass have greater PTH release in response to physiological stimuli (hyperphosphatemic diet or modest hypocalcemia) than osteoporotic patients [4,5]. In contrast, several studies have demonstrated increased bone mass in patients with chronic postsurgical hypoparathyroidism, (PhPT) [6,7]. However, to date no study has addressed fracture susceptibility in patients with PhPT [6,8].

Although the risk of fracture increases significantly with decreasing BMD, large observational studies have demonstrated that osteoporotic fractures can occur across a wide spectrum of BMDs [9,10]. Most likely, these events are related not only to bone mass quantity but also to bone quality, a component not captured by DXA measurements [10,11]. Great efforts are being currently devoted to the determination of clinical conditions associated with a high fracture risk despite an apparent protection by normal or increased BMD. Algorithms that combine BMD with identifiable independent risk factors to estimate a 10-year absolute fracture risk are now being used to provide a more holistic evaluation of absolute fracture risk over a 10-year period [12]. Attempts to further refine the algorithms have been made by taking into account novel variables such as type 2 diabetes mellitus, cardiovascular disease, asthma and use of tricyclic antidepressants in addition to the traditional variables of fracture risk assessment (FRAX) [13]. In addition, the use of other specific bone sites which are largely assessed by radiological exams can potentially be used for the early recognition of osteoporosis [14]. Approximately 1 in 3 of all radiological examinations is made by dentists [15]. Dental panoramic radiographs provide images of the jaws and there is evidence that jaw BMD and radiomorphometric indices developed for this site can be potentially used for the preventive diagnosis of osteoporotic patients [16,17] and of fracture susceptibility [18]. The OSTEODENT study suggested that an automatic measurement of mandible cortical bone thickness on panoramic radiographs is a valid test for the diagnosis of osteoporosis in women aged 45 to 70 years [18]. Bone mass of the mandible have never been evaluated in patients with chronic PhPT.

The present study was designed to evaluate BMD (lumbar spine, total hip, femoral neck, 1/3 radius and total body), vertebral subclinical fracture and cortical thickness in the inferior region of the mandible in a homogenous group of patients with chronic PhPT. All patients were women previously submitted to thyroidectomy due to atoxic benign multinodular goiter. Additionally, we assessed serum levels of insulin-like growth factor (IGF-I), receptor activator of nuclear factor-κB ligand (RANK-L), osteoprotegerin (OPG), 25-hydroxyvitamin D (25-OH-D), and bone remodeling markers [serum osteocalcina (OC) and urinary deoxypyridinoline (DPD)].

### Subjects and methods

#### Subjects

The study was conducted on 33 postmenopausal women whose clinical characteristics are shown in Table 1. The study group included 16 patients with PhPT (PhPTG) followed up at the Outpatient Clinic of Osteometabolic Disorders of the University Medical Center, School of Medicine of Ribeirão Preto, University of São Paulo (FMRP-USP). The diagnosis of permanent PhPT was established by the concomitant presence of low circulatory levels of calcium and PTH after thyroidectomy, as well as by the requirement of continued calcium and vitamin D treatment to maintain serum calcium levels within the normal range.

**Table 1:** Clinical characteristics and biochemical evaluation of control subjects (CG) and of patients with postsurgical hypoparathyroidism (PhPTG)

	**CG (n = 17)**	**PhPTG (n = 16)**	**Estimated difference**	**p-value**
			**[C.I.(95%)]**	
Age (year)	58.0 ± 6.1	62.3 ± 8.9	−4.31(−9.69;1.06)	*P* = 0.2
Weight(kg)	71.8 ± 13.7	72.6 ± 10.9	−0,82(−9.63;7.98)	*P* = 0.9
Height (m)	1.58 ± 6.4	1.54 ± 7.9	4.18(−0.95;9.31)	*P* = 0.8
BMI (kg/m^2^)	28.5 ± 5.5	30.3 ± 4.2	−1.84(−5.33;1.65)	*P* = 0.4
Duration of PhPT	-	15.3 ± 12.4		
Total calcium (mmol/L)	2.43 ± 0.13*	2.05 ± 0.21	1.45(0.95;1.96)	*P* <0.0001
Ca X Pi	3.11 ± 0.63	3.21 ± 0.59	−1.15(−6.51;4.21)	*P* = 0.50
Albumin (g/L)	44.1 ± 2.4*	42.3 ± 1.8	0.18(0.02;0.33)	*P* < 0.05
Phosphorus (mmol/L)	1.29 ± 0.22*	1.57 ± 0.32	−0.86(−1.46;-0.27)	*P* < 0.05
Alkaline phosphatase (U/L)	235.4 ± 65.8	198.1 ± 37.14	37.29(−1;75.58)	*P* = 0.09
Creatinine (μmol/L)	57.96 ± 7.63*	69.39 ± 21.35	−0.30(−0.15;-0.005)	*P* < 0.05
PTH (ng/L)	42.0 ± 18.7*	5.9 ± 4.6	36.05(26.23;45.88)	*P* < 0.0001
25-hydroxyvitamin D (nmol/L)	82.0 ± 35.02	100.8 ± 31.47	−18.9(−42.6;4.85)	*P* = 0.11
IGF-I (μg/L)	68.76 ± 43.55	51.94 ± 19.15	16.83(−7.33;40.98)	*P* = 0.53

The control group (CG) consisted of 17 adult women, matched to the PhPTG for age, weight, height and body mass index (BMI) (Table 1). The general exclusion criteria established for both groups were a personal or family history of osteometabolic disorders, alcohol abuse, smoking, hepatic or renal disease, or using medications known to interfere with mineral metabolism (anabolic steroids, glucocorticoids, anticonvulsants, diuretics or medication for the treatment of osteoporosis). Additionally, no woman, in both group had premature ovarian failure. The study was approved by the Ethics Committee of the School of Medicine of Ribeirão Preto, University of São Paulo (9523/2008). All subjects gave written informed consent.

The PhPT group had been on treatment for 15.3 ± 12.4 years, with daily intake of elemental calcium, calcitriol and L-thyroxin of 1713 ± 419 mg, 0.33 ± 0.18 μg and 99.3 ± 42.1 μg, respectively. The treatment target was to maintain serum total calcium in the lower limit of normal reference values and to keep serum levels of phosphorus and TSH in the normal range. We excluded from this group all individuals with a history of hyperthyroidism and those with a diagnosis of thyroid cancer, as well as individuals with a diagnosis of pseudohypoparathyroidism and autoimmune hypoparathyroidism.

## Methods

Blood and urine collections and radiographic documentation were performed on two days. On the first day, blood and second void urine were samples collected in the morning between 7:00 and 9:00 after a 10–12 hour fast and kept in an ice-chilled container until centrifugation. Serum aliquots were stored at −70°C until assessment, with all determinations being carried out in the same assay. Subsequently, the volunteers were taken to the Image Center of the University Hospital, FMRP-USP, for DXA (dual energy X-ray absorptiometry) scan in total body, lumbar spine, proximal femur and forearm and standard thoracic and lumbar spine X-rays. On the second day, the individuals went to the Radiology Service of the Dental School of Ribeirão Preto, USP, to perform digital panoramic radiography.

Serum levels of calcium, phosphorus, alkaline phosphatase and creatinine were determined with an automatic biochemical analyzer (KoneLab 6.0, Winer, Argentina) on the day of blood collection. Serum IGF-I was measured by enzyme immunoassay [intra-assay coefficient of variation (CV) = 9.5%; Immunodiagnostic Systems, Fountain Hills, AZ]. Also, serum OC (CV = 5.6%; Quidel, San Diego, CA, USA), 25-OH-D (CV = 7.2%; Immunodiagnostic System, Fountain Hills, AZ), OPG (CV = 5.6%; Quidel, San Diego, CA, USA) and RANKL (CV = 6.8%; Biomedica, Austria) and urinary DPD (CV = 6.7%; Quidel, San Diego, CA, USA), were measured by enzyme immunoassay. Serum PTH was measured by chemiluminescence (CV = 4.7%; SIEMENS, Los Angeles, CA, USA).

### Bone mineral density

BMD was measured by DXA at the femoral neck, total hip, lumbar spine (L1-L4) and distal radius by a trained radiologic technician (Hologic 4500 W densitometer, Hologic Inc., Bedford, MA, USA). *In vivo* precision was 1.2% for L1-L4, 2.3% for femoral neck, 2.7% for total hip and 1.7% for 1/3 radius, respectively. BMD values were expressed as g/cm^2^ and T-Score.

### Vertebral morphometry

Lateral thoracic and lumbar spine radiographs were taken for each patient and images were digitized using a Vidar DiagnosticPro scanner with a spatial resolution of 84 m and the gray scale represented by 8 bits/pixel. Six points were marked with electronic calipers on each of the 13 vertebral bodies from T4 to L4 using K-PACS Workstation software, v 1.6.0. These points were positioned on the outer edge of the upper and lower endplates. Using the six points, the anterior, middle and posterior heights were determined for each vertebral body in millimeters. Image analysis was carried out by consensus between 2 radiologists, both performed by blind assessment. A senior musculoskeletal radiologist reviewed all images in which any of the measured vertebral height ratios in a single film was <0.75 and also solved difficult cases when any doubt was present regarding the positioning of the calipers or to check if the encountered deformity was related to pathologies other than insufficiency fractures. The last analysis was also performed by blind assessment.

The criteria of vertebral deformity were: 1) more than a 20% reduction between two of the above measures and/or a 20% reduction in the relation between any one of the above measurements and the measurement of the corresponding region of the vertebral body immediately superior or inferior to each vertebral body analyzed, or 2) a reduction ≥ 4 mm in the absolute vertebral height value in relation to the other heights in the same vertebral body and in relation to the similar height in the immediate neighboring vertebral bodies.

### Panoramic radiograph

All panoramic radiographs were obtained with an RX Veraviewepocs Digital Panoramic instrument (J. Morita Co). Digital images were examined with RADIOIMP software (Radiomemory, Brazil). In this panoramic images, the thickness of the inferior cortical region of the mandible bone were measured on both sides and in two regions (mental and goniac). The digital panoramic radiograph was inserted into the software and calibrated using magnification (30%) and resolution (300 d.p.i.) factors [19]. The projected areas of interest are used for the determination of the Mental Index and Goniac Index, as previously described [20,21].

### Statistical analysis

All statistical analyses were performed using SAS 9.0 software (SAS/STAT® User’s guide version 9.0, Cary, NC, USA: SAS Institute Inc., 2002). The Student t–test was applied using the PROC *T* TEST of the SAS 9.0 software to compare the results of the two groups. To determine the association of the variables of interest (BMD with hypoparathyroidism duration, IGF-I and 25-OH-D, simple linear regression models were adjusted, yielding the regression coefficients and R-square, through the REG procedure of the SAS 9.0 software. Considering the method described by Hsieh et al. [22] for determining the sample size required for the estimation of a correlation coefficient and for linear regression models, a sample of size 16 is sufficient for estimating a correlation coefficient equal to 0.65 with a level of significance of 0.05 and a power of 0.20. Thus, it was established that the sample size in each group should be at least 16. Data are expressed as mean ± SD.

## Results

Table 1 shows that both groups were appropriately matched for age (range: CG = 49–70 vs PhPTG = 45–77 years), weight (range: CG = 46-94.6 vs PhPTG = 54.8–98.5 Kg) height (range: CG = 1.47–1.74 vs PhPTG = 1.47–1.69 m), BMI (range: CG = 16.9–36.1 vs PhPTG = 23.3–38.0 kg/m^2^). The duration of postsurgical hypoparathyroidism ranged from 2 to 44 years (mean = 15.3 ± 12.4 years). Both groups presented normal serum creatinine levels (CG = 57.96 ± 7.63 μmol/L vs PhPTG = 69.39 ± 21.35 μmol/L). All volunteers had creatinine clearance above 60 ml/min. The serum levels of TSH were normal in PhPT group (PhPTG = 2.6 ± 1.0 mIU/L).

Serum levels of total calcium (CG = 2.43 ± 0.13 vs PhPTG = 2.05 ± 0.21 mmol/L; *P* < 0.0001), total calcium corrected by albumin (CG = 2.39 ± 0.21 vs PhPTG = 2.01 ± 0.26 mmol/L, *P* < 0.005), as well as PTH (CG = 4.45 ± 1.99 vs PhPTG = 0.63 ± 0.49 pmol/L, *P* < 0.0001) were significantly lower in PhPTG. The control group showed significantly lower serum phosphorus levels than PhPTG (CG = 1.29 ± 0.22 vs PhPTG = 1.57 ± 0.32 mmol/L, *P <* 0.01). The groups showed nonsignificant differences in circulatory levels of 25-OH-D and IGF-I (Table 1).

Circulatory levels of the biochemical marker of bone formation, OC, were significantly lower in PhPTG (6.75 ± 2.36 μg/L) than in CG (9.64 ± 2.54 μg/L), *P* < 0.01, whereas there was no significant difference in urinary excretion of DPD (CG = 7.44 ± 3.0 vs PhTHG = 11.15 ± 13.16 nmol/mmol creatinine; p = 0.77). Both groups presented similar serum levels of RANKL (CG = 0.26 ± 0.21 vs PhPTG = 0.29 ± 0.27 pg/dl; p = 0.57) and OPG (CG = 0.094 ± 0.012 vs PhPTG = 0.1 ± 0.025 pmol/L; p = 0.44).

**Figure 1:**
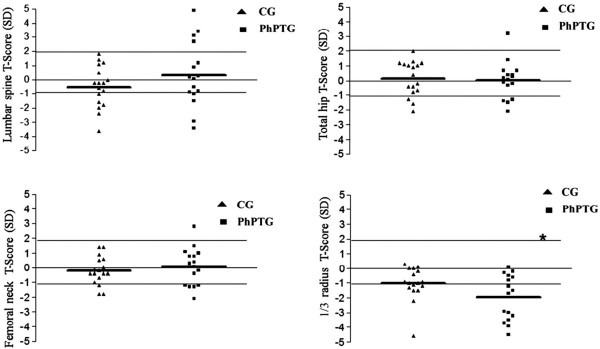
Distribution of bone mineral density values of lumbar spine (A), femoral neck (B), total hip (C) and 1/3 d forearm (D) of the control group (CG) and of the postsurgical hypoparathyroidism group (PhPTG).

Total body BMD was slightly higher in PhPTG (0.923 ± 0.13 g/cm^2^) than in CG (0.904 ± 0.10 g/cm^2^). The mean values of lumbar spine BMD did not differ significantly between groups (CG = 0.970 ± 0.15 g/cm^2^ vs PhPTG = 1.093 ± 0.26 g/cm^2^). However, there was wide heterogeneity of T-score distribution within PhPTG, with 4 patients presenting a high T-score (> + 2.0 SD), 9 patients presenting a normal T-score (between +2 and −1.0 SD), 1 patient presenting a T-score compatible with osteopenia, and 2 patients presenting L1-L4 BMD compatible with osteoporosis (T-score < −2.5 SD) (Figure 1). Lumbar spine BMD was also evaluated after exclusion of the dominant vertebral body when there was a difference higher than 1 SD between two contiguous vertebral bodies. The results obtained in this second evaluation did not differ significantly from those obtained in the first evaluation (CG = 0.970 ± 0.153 vs PhPTG = 1.088 ± 0.250 g/cm^2^). Both groups showed similar BMD values in femoral neck (CG = 0.826 ± 0.111 vs PhPPG = 0.865 ± 0.159 g/cm^2^) and total hip (CG = 0.961 ± 0.145 vs PhPTG = 0.953 ± 0.162 g/cm^2^) (Figure 1). However, BMD values for the 1/3 radius were significantly lower in PhPTG compared to control group (CG = 0.630 ± 0.07 vs PhPTG = 0.570 ± 0.09, *P* < 0.05) (Figure 1).

Table 2 shows that 10 of the 16 PhPTG patients (63%) had morphometric vertebral fractures, as opposed to only 2 CG individuals. The table also shows that morphometric vertebral fractures were not restricted to individuals with a low bone mass. Actually, 3 of the 4 patients with a BMD T-score above + 2SD had subclinical fractures in the thoracic and/or lumbar spine.

**Table 2:** Number of vertebral fractures, bone mineral density (T-score) and mental index in the control group (CG) and postsurgical hypoparathyroidism group (PhPTG)

**CG**	**Vertebral fracture**	**T-score (L1-L4)**	MI *	**PhTPG**	**Vertebral fracture**	**T-score (L1-L4)**	**MI**
**1**	0	−2	3.02	**1**	0	2.7	3.36
**2**	0	1.2	3.59	**2**	0	0.3	2.80
**3**	0	0.0	3.60	**3**	0	−0.8	1.82
**4**	0	−1.8	3.32	**4**	3 (T6, T7, T11)	0.2	3.35
**5**	4 (T5, T6, T7, T11)	1.8	3.9	**5**	6 (T6, T8, T9, T10, T11, T12)	3.1	3.09
**6**	0	−1.6	3.48	**6**	3 (T6, T8, T11)	−3.4	2.32
**7**	2 (T5, T6)	−0.6	3.37	**7**	0	−0.9	2.20
**8**	0	−3.6	1.66	**8**	3 (L1, L3, L4)	−0.5	3.50
**9**	0	−0.2	4.44	**9**	1 (T5)	1.2	3.30
**10**	0	−1	4.04	**10**	0	−1.5	2.26
**11**	0	−2.4	3.25	**11**	8 (T7, T8, T10, T11, L1, L2, L3, L4)	3.4	4.22
**12**	0	−0.2	2.74	**12**	1 (T11)	4.9	3.35
**13**	0	0.5	3.54	**13**	1 (L4)	−1	2.02
**14**	0	1.1	3.30	**14**	0	0.1	3.44
**15**	0	−0.8	4.21	**15**	2 (T7, T8)	−2.9	2.05
**16**	0	1.4	2.81	**16**	1 (T11)	0.9	1.38
**17**	0	−0.2	4.39	**-**	-	-	-
Mean ± SD	-	−0.49 ± 1.47	3.45 ± 0.68	-	-	0.36 ± 2.28	2.79 ± 0.78

*Indicates a significant difference between groups, *P* < 0.05.

**Figure 2:**
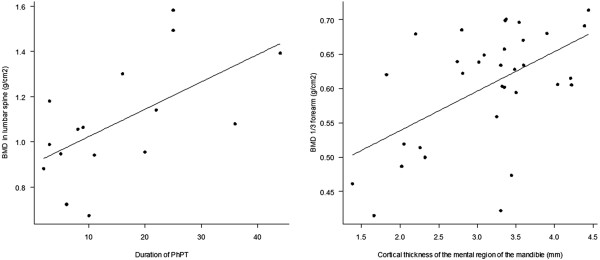
(Right) Correlation between bone mineral density in lumbar spine and duration of postsurgical hypoparathyroidism (p = 0.03 adjusted by TSH; R2 = 0.35) and (Left) correlation between bone mineral density and cortical thickness of the mental region of the mandible (p < 0.01; R2 = 0.30).

There was a significant correlation between the duration of postsurgical hypoparathyroidism and lumbar spine BMD (Figure 2). However, there was no correlation between the duration of postsurgical hypoparathyroidism and vertebral fracture. There was no correlation between BMD and serum IGF-I levels or between BMD and serum 25-OH-D levels.

There was no significant difference between groups regarding inferior cortical thickness in the region of the mandible angle (goniac). Inferior cortical thickness in the mental mandible was significantly decreased in PhPTG (Table 2). There was no significant correlation between lumbar spine BMD and cortical thickness in the mental region of the mandible. However, this last index was significantly and positively correlated with 1/3 distal forearm BMD (Figure 2).

## Discussion

Skeletal fracture has not been considered a potential problem in postsurgical hypoparathyroidism, especially after clinical investigation, based on surrogate parameters, have shown that BMD is preserved or even enhanced in PhPT [7,23]. The present study demonstrated that patients with PhPT have marked heterogeneity in lumbar spine BMD, usually exhibiting high or normal bone mass, while densitometric osteoporosis is uncommon. Paradoxically, we have observed a high frequency of morphometric fracture of lumbar and thoracic vertebrae in patients with PhPT, which is not restricted to those with low bone mass. A previous nonvertebral fracture was present in one patient of the PhPT group, while no individual of the control group sustained clinical fracture. In addition, our results show that panoramic radiography of the mandible is a useful tool for the recognition of bone disorders in PhPT. These results are of great significance in the clinical setting since these data correspond to the first evidence of increased risk of fractures in postsurgical hypoparathyroidism. Therefore, these preliminary results encourage further investigation of the etiopathogenesis and prevalence of this condition and a more appropriate approach to the management of this particular group of patients.

There are studies showing a differential impact of PhPT on diverse bone sites, the increase in BMD being higher in the lumbar spine than in the proximal femur [23], whereas forearm BMD was not positively affected [24]. For Instance, Duan et al. evaluated 10 postmenopausal women harboring postsurgical hypoparathyroidism. The duration of PhPT in this group varied from 0.5 to 51 yr. These authors observed that BMD in patients with hypoparathyroidism was higher when compared with the age predicted mean at the lumbar spine and proximal femur, but not at the distal radius [25]. The present study replicates this spectrum on a different level, with BMD being only slightly increased in the lumbar spine due to wide heterogeneity among patients and being modestly decreased in the proximal femur of PhPT patients, whereas bone mass was decreased in the distal forearm. The divergences between studies are probably due to differences in the profile of the patients examined. Different from other studies, we adopted more rigid criteria for the selection of volunteers. Patients with previous diagnosis of thyroid cancer and hyperthyroidism were excluded, ultimately avoiding the inclusion of individuals affected by other disorders associated with bone loss and fracture. Another difference is that previous studies included pre- and postmenopausal women, whereas only postmenopausal women participated in the present study. Akin to other studies, our patients showed a great variability in the duration of PhPT [25]. This aspect can, at least in part, account for the large variation of BMD values in patients with PhPT. The relevance of this point is reinforced by the strong positive correlation between PhPT duration and BMD.

It should be mentioned that BMD assessment by DXA in PhPT was comparable to that described in primary hyperparathyroidism. It is well known that cortical bone is preferentially affected by primary hyperparathyroidism, and the same pattern was observed in PhPT. It should be pointed out that the control group also had distal forearm BMD within the osteopenia range, a fact possibly related to population characteristics, since the same profile was observed in normal individuals in previous studies performed in our laboratory [25]. However, PhPT patients showed significantly lower distal forearm BMD compared to control individuals. Furthermore, the panoramic radiography of the mandible was especially useful to capture the reduction in cortical thickness in another bone site.

Despite the BMD of the lumbar spine, 63% of patients with PhPT showed at least one morphometric fracture in the dorsal/lumbar spine, and morphometric fractures were also detected in patients showing high bone mass. No study has previously evaluated fracture susceptibility in PhPT. The results indicate deterioration of bone quality, the other component that assures bone strength. A previous study [6] detected by Fourier transform infrared imaging of transiliac biopsies that the collagen cross-link ratio was significantly higher in hypoparathyroid subjects compared to normal individuals. Based on these and other results the authors suggested that bone has low turnover rate and acquires a mature or hypermature profile in PhPTH [6]. Although, we have assessed only osteocalcin and DPD as biochemical bone markers of bone remodeling, our results support previous data obtained by bone histomorphometry. Decreased osteocalcin reflects reduced osteoblast activity in hypoparathyroidism. A reduced rate of bone remodeling which was reversed by intermittent administration of subcutaneous PTH has also been demonstrated by histomorphometric analysis in PhPT patients [7]. In support of these data we observed that osteocalcin levels were significantly lower in PhPT patients than in normal individuals. On a long-term basis, the impairment of bone renewal is implicated in inefficient repair of microdamage and ultimately in fracture susceptibility.

Other factors must be taken into account regarding impairment of bone strength in PhPT patients, especially in those with high BMD. Thickening of trabecular bone could compromise bone strength by reducing resilience [26,27]. In normal bone, the elastic properties of trabecular bone allow the skeleton to absorb energy by deforming reversibly when loaded [28]. Although the greater mineral content in the hypoparathyroid bone might produce greater material stiffness, it may do so at the expense of the ability of the bone to deform and thus to absorb and dissipate energy. Without elastic deformation, it is possible that hypoparathyroid bone could be vulnerable to structural failure. Because the effectiveness of bone in stopping cracks is directly proportional to the stiffness ratio across its internal interfaces, a homogeneous material will be less effective in slowing or stopping cracks initiated in the bone matrix, permitting cracks to grow more quickly to critical size and ultimately increase fracture risk [29,30].

Previous studies have shown that growth hormone deficiency impairs PTH circadian rhythm [31] and PTH action [32,33]. In order to detect hormonal alterations in the growth hormone (GH)/IGF-I axis, in the present study we assessed serum IGF-I levels and the results showed that PhPT patients have a lightly lower circulatory IGF-I level than control individuals. However, we did not measure GH secretion or GH/IGF-I sensitivity in PhPT patients. Additionally, vitamin D sufficiency was evaluated and all patients showed normal serum 25(OH)D levels conferring a convenient substrate supply for paracrine/autocrine synthesis of 1,25(OH)_2_D. Endocrine levels of 1,25(OH)_2_D are largely dependent of PTH secretion, but all patients were taking physiological doses of calcitriol. Although, no woman of the control group was receiving calcium and vitamin D supplementation, they exhibited normal serum levels of 25-OHD. In agreement with these results, we did not observe a high frequency of hypovitaminosis D in this region of the state of São Paulo, Brazil, in previous studies [34,35]. As vitamin D deficiency is associated with worse bone outcome, it is likely that our groups were in advantageous conditions compared to other populations with a higher incidence of vitamin D deficiency [36].

Previous studies have shown increased serum levels of RANKL and maintained OPG levels in primary hyperparathyroidism [37]. Also, it was observed that intermittent administration of PTH to glucocorticoid-treated patients increases serum levels of RANKL and decreases serum levels of OPG [38]. In the present study no difference was detected in RANKL and OPG serum level of PhPT patients in comparison to control subjects. To our knowledge, no previous study addressed serum RANKL levels in postsurgical hypoparathyroidism. Although low PTH levels might be expected to provoke decreased RANKL levels, it should be considered that 1,25(OH)_2_D stimulates RANKL synthesis.

The present study has some limitation; the number of patients studied was insufficient to definitively associate postsurgical hypoparathyroidism with increased fracture risk. However, these are important preliminary data for a more comprehensive study to examine the dissociation between bone fragility and bone mineral density in patients harboring postsurgical hypoparathyroidism.

## Conclusions

Our results suggest that PhPT has a great impact on bone structure which is not necessarily detected by BMD density. Subclinical vertebral fracture was identified in more than 60% of PhPT patients, including those exhibiting high BMD. Panoramic radiography of the mandible should be thoroughly scrutinized to determine its place in the diagnosis or screening of osteoporosis. This site was useful to reveal cortical changes in PhPT. Panoramic radiography has the additional advantage of relying on a dentist for the screening of osteoporotic patients. The study encourages further investigation to determine the role of PTH as hormone replacement therapy in postsurgical hypoparathyroidism.

## Abbreviations

BMD: Bone mineral density; BMI: Body mass index; CG: Control group; CV: Coefficient of variation; DPD: Urinary deoxypyridinoline; DXA: Dual energy X-ray absorptiometry; FMRP-USP: School of Medicine of Ribeirão Preto, University of São Paulo; IGF-I: Insulin-like growth factor; OPG: Osteoprotegerin; OC: Osteocalcin; PTH: Parathyroid hormone; PhPT: Hypoparathyroidism; PhPTG: Hypoparathyroidism group; RANK-L: Receptor activator of nuclear factor-κB ligand; 25-OH-D: 25-hydroxyvitamin D.

## Competing interests

The authors declare no conflict of interests.

## Authors’ contributions

MLM participated in patient selection/sample collections, laboratory measurements and data analysis. FAP participated in sample collections, laboratory and bone mass measurerements. MHN-B participated in the study design and in the supervision of vertebral morphometry evaluation. LMM performed radiographic exams and the first blind evaluation of vertebral morphometry. SRT performed radiographic exams and the second blind evaluation of vertebral morphometry. PCAW performed radiographic exams of mandible and the correspondent evaluations. LMZM participated in patient selection, study design and laboratory evaluations. FJAdP participated in study design and laboratory measurements, supervised data collection and analysis and wrote the manuscript. All authors revised the manuscript. All authors read and approved the final manuscript.

## Pre-publication history

The pre-publication history for this paper can be accessed here:

http://www.biomedcentral.com/1472-6823/13/1/prepub
